# Intranasal Ketamine for Depression in Adults: A Systematic Review and Meta-Analysis of Randomized, Double-Blind, Placebo-Controlled Trials

**DOI:** 10.3389/fpsyg.2021.648691

**Published:** 2021-06-01

**Authors:** Dongjiao An, Changwei Wei, Jing Wang, Anshi Wu

**Affiliations:** Department of Anesthesiology, Beijing Chaoyang Hospital, Capital Medical University, Beijing, China

**Keywords:** intranasal, ketamine, unipolar disorder, depression, meta-analysis

## Abstract

**Background:**

There is growing interest in glutamatergic agents as a treatment for depression, especially intranasal ketamine, which has become a hot topic in recent years. We aim to assess the efficacy and safety of intranasal ketamine in the treatment of major depressive disorder (MDD), especially treatment-resistant depression (TRD).

**Methods:**

We searched Medline, EMBASE, and the Cochrane Library until April 1, 2020 to identify double-blind, randomized controlled trials with allocation concealment evaluating intranasal ketamine in major depressive episodes. Clinical remission, response, and depressive symptoms were extracted by two independent raters. The outcome measures were Montgomery–Asberg Depression Rating Scale (MADRS) score improved from baseline, clinical response and remission, dissociative symptoms, and common adverse events. The analyses employed a random-effects model.

**Results:**

Data were synthesized from five randomized controlled trials (RCTs) employing an intranasal esketamine and one RCT employing intranasal ketamine, representing 840 subjects in parallel arms, and 18 subjects in cross-over designs (*n* = 858 with MDD, *n* = 792 with TRD). The weighted mean difference of MADRS score was observed to decrease by 6.16 (95% CI 4.44–7.88) in 2–4 h, 9.96 (95% CI 8.97–10.95) in 24 h, and 4.09 (95% CI 2.18–6.00) in 28 day. The pooled relative risk (RR) was 3.55 (95% CI 1.5–8.38, *z* = 2.89, and *p* < 0.001) for clinical remission and 3.22 (95% CI 1.85–5.61, *z* = 4.14, and *p* < 0.001) for clinical response at 24 h, while the pooled RR was 1.7 (95% CI 1.28–2.24, *z* = 3.72, and *p* < 0.001) for clinical remission and 1.48 (95% CI 1.17–1.86, *z* = 3.28, and *p* < 0.001) for clinical response at 28 day. Intranasal ketamine was associated with the occurrence of transient dissociative symptoms and common adverse events, but no persistent psychoses or affective switches.

**Conclusion:**

Our meta-analysis suggests that repeated intranasal ketamine conducted a fast-onset antidepression effect in unipolar depression, while the mild and transient adverse effects were acceptable.

**Systematic Review Registration:**

PROSPERO, CRD42020196856.

## Introduction

Major depressive disorder (MDD) is a common but severe psychiatric condition, which exerts a serious impact on health by increasing suicidal thoughts and behaviors. In 2017, the prevalence of MDD was estimated to be 7.1% (about 17 million adults) in the United States (Kryst et al., 2020), which has been an increasing trend in recent years. However, the effects of treatment for MDD are not satisfactory. Approximately 30% of patients are considered to have treatment-resistant depression (TRD; Rush, 2011), which is usually defined as lack of response to at least two anti-depressive monotherapies of adequate dose and duration, including the current episode (Souery et al., 2006). Therefore, it is necessary to explore a more effective and rapid-onset anti-depressive drug.

Ketamine, the glutamate *N*-methyl-D-aspartate (NMDA) receptor antagonist, is a traditional and widely used anesthetic drug (Zanos et al., 2018). In 2000, Berman et al. demonstrated that intravenous sub-anesthetic dose of ketamine showed a rapid anti-depressive effect (Berman et al., 2000). Subsequently, several randomized controlled trials (RCT) studies have confirmed the efficacy of intravenous ketamine in anti-depressive therapy (Zarate et al., 2006; Murrough et al., 2013; Phillips et al., 2019). A series of meta-analyses summarized the results of RCTs and confirmed the rapid and transient anti-depressive effect of intravenous ketamine (Caddy et al., 2015; McCloud et al., 2015). Therefore, ketamine has emerged as a novel treatment for patients with MDD, especially TRD. However, the inconvenience of intravenous administration plagues depressed patients who require prolonged treatment and psychiatrists who proceed with long-term observation. In 2014, Lapidus et al. began to explore a new and convenient route of intranasal administration. As expected, intranasal ketamine administration was highly effective in the amelioration of depressive symptoms and significantly reduced the Montgomery–Asberg Depression Rating Scale (MADRS) score. Headache, dizziness, or dissociative symptoms were common and transient adverse events, which are similar to intravenous delivery (Lapidus et al., 2014).

The purpose of our research was to evaluate the efficacy, safety, and tolerability of intranasal ketamine in the treatment of MDD, especially TRD.

## Methods

### Search Strategy

We identified articles for inclusion in this meta-analysis by searching Medline, EMBASE, and the Cochrane Library until April 1, 2020. Key words such as “depressive disorder,” “major depressive disorder,” “ketamine,” and “randomized controlled trial” with their various relevant combinations were used as title/abstracts for the literature search. Study authors were mailed for literature without full-text or other useful information. Studies that had not been fully published (e.g., conference abstract) or without full-text were excluded. The search procedure is described in detail in the Supplementary Material.

### Study Selection

Studies were included if they satisfied all the following criteria: (1) study validity: random allocation; allocation concealment; double-blind; placebo-controlled; parallel or cross-over design; clinician-rated primary outcome measure; and ≥10 subjects total number. (2) Sample characteristics: subjects (age ≥ 18 years) with a clear diagnosis of a primary major depressive episode (only unipolar) according to DSM-IV criteria. (3) Treatment characteristics: intranasal administration of ketamine or esketamine (use in combination with other antidepressants was permitted). (4) Publication had to be written in English.

**FIGURE 1:**
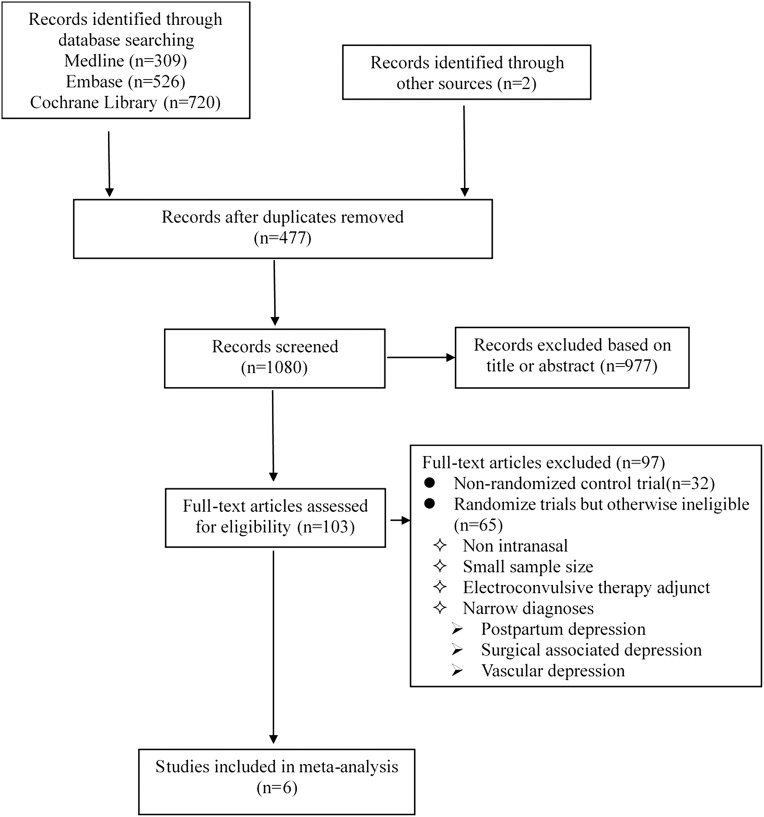
Flow diagram for systematic review.

Exclusion criteria: (1) “narrow” diagnoses (e.g., postpartum depression, surgical associated depression); secondary depression (e.g., vascular depression). (2) Ketamine as an electroconvulsive therapy adjunct. A summary of the selection process is given in Figure 1.

### Data Extraction

Data recorded by two independent observers were extracted from studies meeting the criteria above. The following related data were extracted: (1) characteristics: study, design, age, gender, and sample. (2) Ketamine dose, formulation, and frequency. (3) Control condition: substance, dose, and frequency. (4) Primary outcome measures: depressive symptoms as assessed by MADRS (Williams and Kobak, 2008). (5) Secondary outcome measures: clinical response, clinical remission, record of the primary and secondary outcomes at different times. (6) Safety assessments: psychotomimetic and dissociative symptoms as measured by the Clinician Administered Dissociative States Scale (CADSS; Bremner et al., 1998) and common adverse events. For trials with a cross-over design, we considered only results from the first period prior to cross-over.

### Quality Assessment

Assessment of risk of bias in included studies. Two review authors (DA, JW) independently assessed risk of bias for each study using the criteria outlined in the Cochrane Handbook for Systematic Reviews of Interventions (Higgins et al., 2011). Any disagreements were resolved by discussion or by involving another review author (CW). We assessed the risk of bias according to the following domains.

1. Random sequence generation
2. Allocation concealment
3. Blinding of participants and personnel
4. Blinding of outcome assessment
5. Incomplete outcome data
6. Selective outcome =reporting
7. Other bias

Publication bias was not carried out because the number of included articles did not exceed seven.

### Data Synthesis and Analyses

Analyses were performed using the Statistics/Data Analysis MP-parallel Edition 14.0. We calculated the relative risk (RR) with corresponding 95% confidence interval (95% CI) for dichotomous event-like outcomes, the weighted mean difference (WMD) along with corresponding 95% CI for continuous outcomes. All analyses were performed with a random-effects model (Riley et al., 2011). An effect size was considered significant when the 95% CI excluded 0 and when the *p* value was less than 0.05.

We assessed heterogeneity using *I*^2^ value and two-tailed *p* values, which estimated the amount of total variation attributable to heterogeneity rather than chance. Values of *p* < 0.05 and *I*^2^ > 50% were deemed as indicative of study heterogeneity and sensitivity analysis was needed.

## Results

### Literature Search

Our literature search is detailed in Figure 1 and the search strategies are shown in Supplementary Table 1. Finally, we identified six double-blind RCTs (Lapidus et al., 2014; Canuso et al., 2018; Daly et al., 2018; Fedgchin et al., 2019; Ochs-Ross et al., 2019; Popova et al., 2019) through our systematic review, all of which met the inclusion criteria. Study quality was assessed using the Cochrane Collaboration’s Tool for Assessing Risk of Bias (Higgins et al., 2011; Supplementary Table 2).

### Included RCTs: Main Characteristics

Overall, six RCTs (Lapidus et al., 2014; Canuso et al., 2018; Daly et al., 2018; Fedgchin et al., 2019; Ochs-Ross et al., 2019; Popova et al., 2019) were included in our meta-analysis, totaling 858 subjects with a MDD (*n* = 792 with TRD; Table 1). One of the studies was a crossover RCT (Lapidus et al., 2014), while the rest were parallel arm RCTs (Canuso et al., 2018; Daly et al., 2018; Fedgchin et al., 2019; Ochs-Ross et al., 2019; Popova et al., 2019).

**TABLE 1:** Characteristics of included studies.

Study	Design	Patients	Sample	Intervention	Comparator	Primary outcome	Secondary outcomes
Lapidus et al., 2014	Crossover RCT, DB	21–65 years, MDD, TRD	18	50 mg of racemic ketamine (once per week)	0.9% saline solution	Change from baseline in MADRS total score to 24 h	Response rate at 24 h safety
Canuso et al., 2018	RCT, DB	19–64 years, MDD	66	84 mg of esketamine (56 mg if intolerance) twice weekly for 4 weeks	Placebo	Change from baseline in MADRS total score to 4 h, 24 h, and 25 day	Remission rate at 24 h, 25 day safety
Daly et al., 2018	RCT, DB, phase 2	20–64 years, TRD	67	28, 56, or 84 mg of esketamine (twice weekly)	Water for injection	Change from baseline in MADRS total score to 2, 24 h	Response rate at 24 h, remission rate at 24 h safety
Fedgchin et al., 2019	RCT, DB, phase 3	18–64 years, TRD	346	56 or 84 mg of esketamine (twice per week) plus OA	Placebo plus OA	Change from baseline in MADRS total score to day 28	Response rate at 24 h safety
Popova et al., 2019	RCT, DB, phase 3	18–64 years, TRD	223	56 or 84 mg of esketamine (twice per week) plus OA	Placebo plus OA	Change from baseline in MADRS total score to day 28	Response rate at 24 h, 28 day, remission rate at 28 day safety
Ochs-Ross et al., 2019	RCT, DB, phase 3	≥65 years, TRD	138	28, 56, or 84 mg of esketamine (twice per week, flexible dose) plus OA	Placebo plus OA	Change from baseline in MADRS total score to day 28	Response rate at 28 day, remission rate at 28 day safety

*RCT, randomized controlled trial; DB, double blind; MDD, major depressive disorder; TRD, treatment-resistant depression; and MADRS, Montgomery–Asberg Depression Rating Scale.*

Esketamine was administered intranasally in five studies with different doses and frequencies (Canuso et al., 2018; Daly et al., 2018; Fedgchin et al., 2019; Ochs-Ross et al., 2019; Popova et al., 2019), in another study racemic ketamine was used at 50 mg once a week (Lapidus et al., 2014). Study drugs were provided in a special nasal spray device. Each inhalation should be maintained for a certain period to ensure the effectiveness of inhaled medication. Three studies combined with a newly initiated oral antidepressant (Fedgchin et al., 2019; Ochs-Ross et al., 2019; Popova et al., 2019), which was assigned by the investigator from four choices (duloxetine, escitalopram, sertraline, or venlafaxine extended release) and could not be one that the patient already had non-response to (in the current depressive episode) or had not tolerated.

A bittering agent was added to the placebo formulation to simulate the taste of esketamine in five of the studies (Canuso et al., 2018; Daly et al., 2018; Fedgchin et al., 2019; Ochs-Ross et al., 2019; Popova et al., 2019), while one used 0.9% saline solution (Lapidus et al., 2014), and one used water for injection (Daly et al., 2018). Participants in five studies were younger than 65 years (Lapidus et al., 2014; Canuso et al., 2018; Daly et al., 2018; Fedgchin et al., 2019; Popova et al., 2019), while one study involved patients older than 65 years (Ochs-Ross et al., 2019).

Primary outcome measures were change from baseline to different time in MADRS total score. Earlier studies focused on changes within a week (Lapidus et al., 2014; Daly et al., 2018), while more recent studies extended the observation period to a month or even longer (Canuso et al., 2018; Fedgchin et al., 2019; Ochs-Ross et al., 2019; Popova et al., 2019). Secondary outcome measures were the proportion of individuals meeting the response and remission criteria. Response was defined as a 50% or greater decrease in the MADRS score from baseline (Lapidus et al., 2014; Daly et al., 2018; Fedgchin et al., 2019; Ochs-Ross et al., 2019; Popova et al., 2019), and remission was defined as a MADRS score of ≤9 (Lapidus et al., 2014), ≤10 (Daly et al., 2018), or ≤12 (Canuso et al., 2018; Fedgchin et al., 2019; Ochs-Ross et al., 2019; Popova et al., 2019). In addition, safety was evaluated and major adverse reactions were demonstrated, but no serious adverse reactions occurred.

**FIGURE 2:**
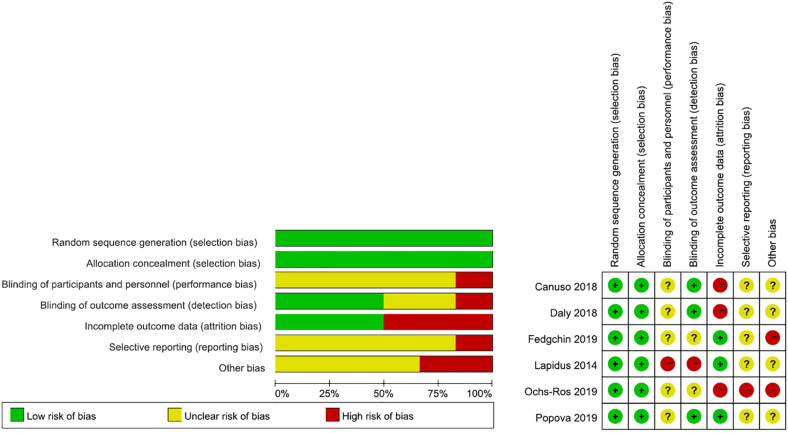
Potential sources of bias in included trials.

A rigorous literature quality evaluation was conducted and potential sources of bias were summarized, as shown in Figure 2.

### Efficacy Results

**FIGURE 3:**
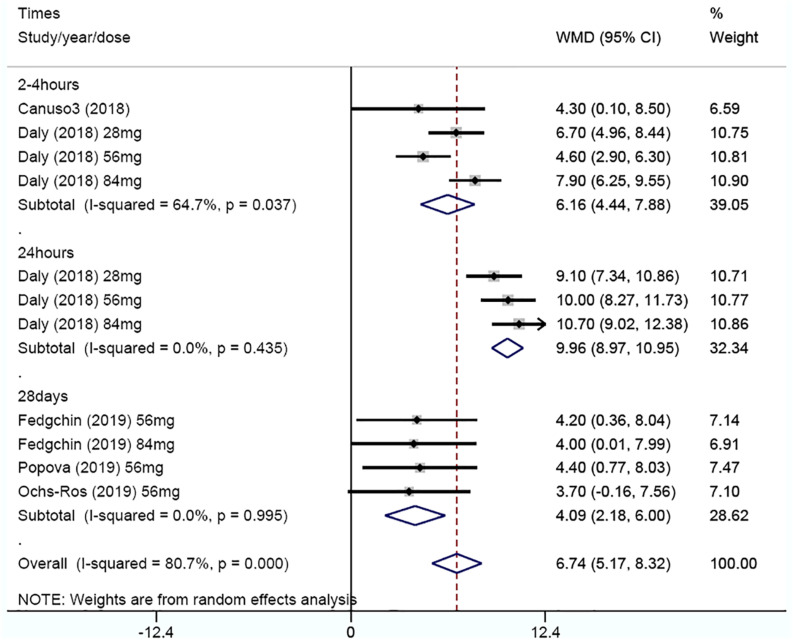
Subgroup analysis of weighted mean difference in MADRS score decreased from baseline after 2–4 h, 24 h, and 28 day.

Effects on depression severity scores over time were represented as the MADRS score decreased (improved) from baseline to any time after the first dose in both the esketamine group and the placebo group. Five of the studies detailed improvements in MADRS scores at different doses and different times (Canuso et al., 2018; Daly et al., 2018; Fedgchin et al., 2019; Ochs-Ross et al., 2019; Popova et al., 2019). In order to facilitate the summary, we conducted subgroup analysis according to 2–4 h, 24 h, and 28 day (Figure 3). Overall, a WMD of 6.74 (95% CI 5.17–8.32, *z* = 8.38, and *p* = 0.00) was observed, indicating a significant difference in outcome favoring ketamine. WMD was observed to be 6.16 (95% CI 4.44–7.88, *z* = 7.02, and *p* = 0.00) in 2–4 h, 9.96 (95% CI 8.97–10.95, *z* = 19.64, and *p* = 0.00) in 24 h, and 4.09 (95% CI 2.18–6.00, *z* = 4.19, and *p* = 0.00) in 28 day. Sensitivity analysis was necessary to complete in 2–4 h because of heterogeneity (*I*^2^ = 64.7%, *P* = 0.037).

The article-by-article elimination method was used for sensitivity analysis, which revealed a relative robustness of the findings, with WMD of 5.47 (95% CI, 3.87 to 7.08, and *P* = 0.00) when study Daly 2018 (84 mg) was excluded, 6.99 (95% CI, 5.53 to 8.45, and *P* = 0.00) when study Daly 2018 (56 mg) was excluded, 5.84 (95% CI, 3.24 to 8.45, and *P* = 0.00) when study Daly 2018 (28 mg) was excluded, and 6.41 (95% CI, 4.50 to 8.31, and *P* = 0.00) when study Canuso 2018 was excluded (Table 2).

**TABLE 2:** Sensitivity analyses for MADRS score decreased from baseline to 2–4 h.

Model	Excluded study	Sample	WMD (95%CI)	*P* value	Heterogeneity	*P* value for heterogeneity
Model 1	None	198	6.16 (4.44,7.88)	0.00	64.70%	0.04
Model 2	Daly 2018 (84 mg)	154	5.47 (3.87,7.08)	0.00	38.00%	0.20
Model 3	Daly 2018 (56 mg)	154	6.99 (5.53,8.45)	0.00	29.00%	0.24
Model 4	Daly 2018 (28 mg)	154	5.84 (3.24,8.45)	0.00	76.00%	0.02
Model 5	Canuso 2018	132	6.41 (4.50,8.31)	0.00	74.00%	0.02

*Model represented inclusion with the studies. Mode 1: Canuso 2018, Daly 2018 (28 mg), Daly 2018 (56 mg), and Daly 2018 (84 mg) included. Mode 2: Canuso 2018, Daly 2018 (28 mg), and Daly 2018 (56 mg) included. Mode 3: Canuso 2018, Daly 2018 (28 mg), and Daly 2018 (84 mg) included. Mode 4: Canuso 2018, Daly 2018 (56 mg), and Daly 2018 (84 mg) included. Mode 5: Daly 2018 (28 mg), Daly 2018 (56 mg), and Daly 2018 (84 mg) included.*

**FIGURE 4:**
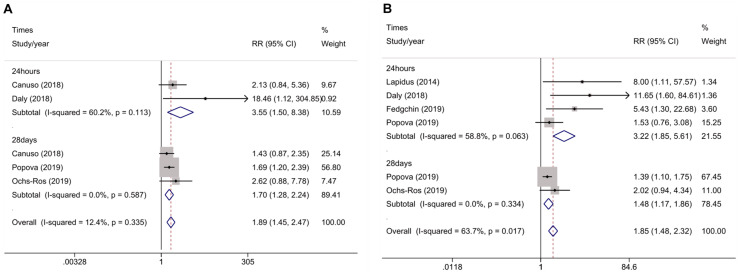
Meta-analysis of rates of clinical remission **(A)** and rates of clinical response **(B)** for ketamine v. placebo in major depression.

Rates of clinical response and remission were available for all RCTs, which were analyzed at 24 h and 28 day (Figures 4A,B). At 24 h, the pooled RR was 3.55 (95% CI 1.5–8.38, *z* = 2.89, and *p* < 0.001) for clinical remission and 3.22 (95% CI 1.85–5.61, *z* = 4.14, and *p* < 0.001) for clinical response, indicating a significant difference in outcome favoring ketamine. While at 28 day, the pooled RR was 1.7 (95% CI 1.28–2.24, *z* = 3.72, and *p* < 0.001) for clinical remission and 1.48 (95% CI 1.17–1.86, *z* = 3.28, and *p* < 0.001) for clinical response, suggesting that ketamine had a significant anti-depressive effect. There were no evidence of heterogeneities in clinical remission (*I*^2^ = 60%, *P* = 0.113) at 24 h and remission (*I*^2^ = 0.00%, *P* = 0.587) at 28 day or clinical response (*I*^2^ = 58.8%, *P* = 0.063) at 24 h and response (*I*^2^ = 0.00%, *P* = 0.334) at 28 day.

### Safety Results

Dissociative symptoms, as measured by the CADSS, were recorded with data in Lapidus’ study (Lapidus et al., 2014). Among ketamine responders, the increase in CADSS score at +40 min was 1.75 ± 4.17 compared to 1.09 ± 1.76 in ketamine non-responders, while dissociative symptoms resolved by +240 min. Although the rest of the studies illustrated the trend, detailed data were not available. Dissociative symptoms generally began shortly after the start of dosing, peaked at 30–40 min after dosing, and resolved within 1.5–2 h (Canuso et al., 2018; Daly et al., 2018; Fedgchin et al., 2019; Ochs-Ross et al., 2019; Popova et al., 2019).

Common adverse events were observed in each study. The incidence of dizziness, dissociation, dysgeusia, vertigo, and nausea seemed to be higher in patients treated with intranasal ketamine or esketamine by forest plot analysis (Table 3). These studies also showed that most of these symptoms resolved a few hours post-administration.

**TABLE 3:** Forest plot results of commonly occurring adverse events.

Adverse events	Sample	RR (95%CI)	*Z* value	*P* value	Heterogeneity	*P* value for heterogeneity
Dizziness	895	3.30 (2.20,4.95)	5.78	0.00	0.00%	0.70
Dissociation	895	5.68 (3.34,9.65)	6.42	0.00	0.00%	0.55
Dysgeusia	895	1.37 (0.99,1.89)	1.88	0.06	0.00%	0.48
Vertigo	895	7.04 (3.56,13.93)	5.61	0.00	0.00%	0.81
Nausea	895	3.25 (2.19,4.84)	5.81	0.00	0.00%	0.56

## Discussion

Research on the anti-depressive effects of ketamine started from intravenous use, and gradually expanded to subcutaneous, oral, intranasal, and other methods in recent years. The medication also shifted from ketamine to esketamine. As early as 2015, Caddy et al. demonstrated the effectiveness of intravenous ketamine at 24 h (random-effects SMD -1.42, 95% CI -2.26 to -0.57) in a meta-analysis (Caddy et al., 2015). Similarly, the WMD of MADRS score was observed to decrease to 6.16 (95% CI 4.44–7.88) in 2-4 h, 9.96 (95% CI 8.97–10.95) in 24 h, and 4.09 (95% CI 2.18–6.00) in 28 day in our study, which exhibited ketamine’s explicit anti-depressive effects. Our results showed a stronger effect on depression than Caddy’s results, due to the difference in the size of the effect chosen, WMD for ours and SMD for Caddy’s. Murrough et al. (2013) reported a mean ketamine-placebo difference of 7.95 (95% CI: 3.20–12.71) on the MADRS scale 24 h following a single dose (0.5 mg/kg) of intravenous ketamine, which was comparable to the improvement in our meta-analysis. In fact, the route of administration may not affect the anti-depressive effect of ketamine (Romeo et al., 2015). Intranasal ketamine had an up to 45% bioavailability, and there were no differences in pharmacokinetics between preparation, including injection (Yanagihara et al., 2003; Li and Vlisides, 2016). It may depend on the actual blood concentration, in which it was proven that 56 and 84 mg intranasal doses of esketamine produce plasma esketamine levels that are in the pharmacokinetic range achieved by intravenous administration of esketamine at 0.2 mg/kg (Singh et al., 2016).

Ketamine is a 1:1 racemic mixture of the S (+) enantiomer (esketamine) and the R (–) enantiomer (arketamine). Esketamine, which antagonizes the glutamatergic NMDA receptor non-competitively and binds to the phencyclidine binding site (Zanos et al., 2018) affecting the glutamate receptor modulation three to four-folds higher than arketamine, is more commonly used in the treatment of MDD (Vollenweider et al., 1997). Ketamine is a short-acting, fast-metabolizing antidepressant that can last up to 7 days after a single dose (Berman et al., 2000), suggesting other mechanisms may be involved. In the metabolism of ketamine (2R,6R)-hydroxynorketamine (HNK), is essential for its anti-depressive effects, which induced a robust increase in α-amino-3-hydroxy-5-methyl-4-isoxazole propionic acid receptor-mediated excitatory post-synaptic potentials (Zanos et al., 2016). However, the results from animal models of depression need to be confirmed in humans. Further clinical studies have shown that ketamine is thought to enhance synaptic plasticity and reverse the synaptic pathophysiology in brain regions associated with depression, and that the prefrontal cortex-related circuit modulation is crucial to the anti-depressive effects of ketamine (Iadarola et al., 2015; Chen et al., 2019; Sumner et al., 2020).

An article expounded that ketamine had different effects between unipolar and bipolar depression, given that people with unipolar depression had on average lower levels of total glutamate and glutamine (Glx) than healthy controls, while the patients with bipolar depression tended toward higher Glx than healthy controls (Taylor, 2014). Another article showed that anterior cingulate glutamate levels were reduced in both unipolar and bipolar depression groups relative to healthy controls, but this only reached significance in the unipolar group (Wise et al., 2018). So, we hypothesized that ketamine might be more specific for unipolar depression, thus the inclusion criteria included unipolar depression only.

Despite the fast-onset anti-depressive effects, intranasal esketamine was associated with undesirable adverse reactions including dizziness, dissociation, dysgeusia, vertigo, and nausea. While the included RCT studies in the present article generally reported acceptable side effects, most of them were mild to moderate in severity, and occurred on the day of administration, then resolved on the same day, because of these side effects and potential abuse, patients should be monitored for hours after administration, and esketamine should be strictly regulated and used with caution. In addition, considering the frequent and prolonged use of ketamine in depression patients, the harmful consequences included neurocognitive impairment, interstitial cystitis, respiratory depression, and liver injury (Singh et al., 2017). Ketamine was demonstrated to have wide-ranging and profound effects on memory, including semantic and episodic memory, short- and long-term memory, while this kind of memory impairment may be reversible after abstinence for a certain time (Morgan and Curran, 2006; Morgan et al., 2010). In a long-term trial (intranasal esketamine administration for up to 52 weeks including a 4-week induction phase and 48-week maintenance phase) Wajs et al. showed that cognitive performance either improved or remained stable post-baseline, and there was no case of interstitial cystitis or respiratory depression. Besides the treatment, emergent dissociative symptoms resolved within 1.5 h post-dose (Wajs et al., 2020). No clinically significant elevation on liver enzymes compared with placebo in the eligible trials contained in this article was reported. In conclusion, current research suggests that long-term esketamine nasal spray had a manageable safety profile (Wajs et al., 2020).

The limitations of our meta-analysis include the limited number of trials and data included in the analyses, which may lead to low statistical power and incomplete results. In particular, the heterogeneity (*I*^2^) could not be completely improved, yet when multiple dimensions, such as dose, time, and article quality of sensitivity analysis were conducted, the ketamine favoring results were relatively robust. We speculated that the reason for the poor effect of the sensitivity analysis might be related to the small number of articles. In addition, the funnel plot to examine publication bias was not drawn for the small number of the included studies (*n* = 6).

In March 2019, intranasal esketamine in conjunction with an oral antidepressant was approved by the Food and Drug Administration for treating TRD in adults. As a new class of antidepressants, esketamine may change the treatment pattern and bring a bright future for people with MDD, especially those with TRD; besides intranasal drug delivery is more convenient and practical for long-lasting therapy. Further studies are needed to investigate the optimal dosage and frequency of drug delivery balancing the efficacy and side effects, and to elucidate if there are any differences in efficacy depending on combined oral antidepressants.

## Conclusion

In summary, the present meta-analysis shows that repeatedly intranasal ketamine conducted a fast-onset antidepression effect in unipolar depression, while the mild and transient adverse effects were acceptable.

## Data Availability Statement

The raw data supporting the conclusions of this article will be made available by the authors, without undue reservation.

## Author Contributions

DA and AW: conceptualization. DA and JW: methodology. DA and CW: data curation and data analysis. DA: draft preparation. AW: supervision. All authors contributed to the article and approved the submitted version.

## Conflict of Interest

The authors declare that the research was conducted in the absence of any commercial or financial relationships that could be construed as a potential conflict of interest.

## Supplementary Material

Supplementary Table 1 Search strategies.

Supplementary Table 2 Cochrane collaboration’s tool for assessing risk of bias of included studies.
