# Supplementary material for: Intranasal Ketamine for Depression in Adults: A Systematic Review and Meta-Analysis of Randomized, Double-Blind, Placebo-Controlled Trials
Source: Front Psychol. 2021 Jun 1;12:648691. doi: 10.3389/fpsyg.2021.648691 (PMC8204747; doi:10.3389/fpsyg.2021.648691)
Supplement: Supplementary Table 1 — Search strategies. [file Table_1.DOCX]

Table S1 Search Strategies

| Name of database | Search Keywords and Results |
| --- | --- |
| 1. Medline | 🞏 Search string using keywords   1. randomized controlled trial.pt. 2. controlled clinical trial.pt. 3. randomized.ab. 4. placebo.ab. 5. clinical trials as topic.sh. 6. randomly.ab. 7. trial.ti. 8. 1 or 2 or 3 or 4 or 5 or 6 or 7 9. exp Ketamine/ 10. Depression/ 11. exp major depressive disorder/ or depressive disorder/ or depressive disorder, major/ or depressive disorder, treatment-resistant/ or dysthymic disorder/ 12. 10 or 11 13. 8 and 9 and 12 |
|  | 🞏 Result 309 publications (inception to 29/Mar/2020) |
| 2. Embase | 🞏 Search string using keywords   1. 'controlled clinical trial'/exp 2. ' Ketamine '/exp 3. 'depression` /exp or 'major depressive disorder` /exp or ' treatment resistant depression` /exp |
|  | 🞏 Result 526 publications (inception to 29/Mar/2020) |
| 3. Cochrane Library | 🞏 Search string using keywords   1. randomized controlled trial.mp. 2. controlled clinical trial.mp. 3. randomized.ab. 4. placebo.ab. 5. 1 or 2 or 3 or 4 6. ketamine/ 7. *"Depression (Emotion)"/ 8. *Major Depression/ 9. *Treatment Resistant Depression/ 10. 7 or 8 or 9 11. 5 and 6 and 10 |
|  | 🞏 Result 720 publications (inception to 29/Mar/2020) |
| Total  (duplicates removed) | 1080/1555 (420 duplicates picked up by Endnote, 57 picked up manually) |
