# Supplementary material for: Intranasal Ketamine for Depression in Adults: A Systematic Review and Meta-Analysis of Randomized, Double-Blind, Placebo-Controlled Trials
Source: Front Psychol. 2021 Jun 1;12:648691. doi: 10.3389/fpsyg.2021.648691 (PMC8204747; doi:10.3389/fpsyg.2021.648691)
Supplement: Supplementary Table 2 — Cochrane collaboration’s tool for assessing risk of bias of included studies. [file Table_2.DOCX]

| Study | Random sequence generation | Allocation concealment | Blinding of participants and personnel | Blinding of outcome assessment | Incomplete outcome data | Selective reporting | Other bias |
| --- | --- | --- | --- | --- | --- | --- | --- |
| Lapidus et al. 2014  Canuso et al. 2018  Daly et al. 2018  Fedgchin et al. 2019  Popova et al. 2019  Ochs-Ross et al. 2019 | +  +  +  +  +  + | +  +  +  +  +  + | -  ?  ?  ?  ?  ? | -  +  +  ?  +  ? | +  -  -  +  +  - | ?  ?  ?  ?  ?  - | ?  ?  ?  -  ?  - |

Table S2 Cochrane Collaboration’s Tool for Assessing Risk of Bias of included studies

Note: “+” (yes), “-” (no), “?” (unclear). High risk was considered when no bittering agent was used.
